# Global Vitamin C Status and Prevalence of Deficiency: A Cause for Concern?

**DOI:** 10.3390/nu12072008

**Published:** 2020-07-06

**Authors:** Sam Rowe, Anitra C. Carr

**Affiliations:** 1 Department of Clinical Sciences, Liverpool School of Tropical Medicine, Liverpool L35QA, UK; sam.rowe@lstmed.ac.uk; 2 Nutrition in Medicine Research Group, Department of Pathology & Biomedical Science, University of Otago, Christchurch 8011, New Zealand

**Keywords:** vitamin C status, hypovitaminosis C, vitamin C deficiency, low and middle income, LMIC, dietary intake, supplement, non-communicable disease, communicable disease, infection

## Abstract

Vitamin C is an essential nutrient that must be obtained through the diet in adequate amounts to prevent hypovitaminosis C, deficiency and its consequences—including the potentially fatal deficiency disease scurvy. Global vitamin C status and prevalence of deficiency has not previously been reported, despite vitamin C’s pleiotropic roles in both non-communicable and communicable disease. This review highlights the global literature on vitamin C status and the prevalence of hypovitaminosis C and deficiency. Related dietary intake is reported if assessed in the studies. Overall, the review illustrates the shortage of high quality epidemiological studies of vitamin C status in many countries, particularly low- and middle-income countries. The available evidence indicates that vitamin C hypovitaminosis and deficiency is common in low- and middle-income countries and not uncommon in high income settings. Further epidemiological studies are required to confirm these findings, to fully assess the extent of global vitamin C insufficiency, and to understand associations with a range of disease processes. Our findings suggest a need for interventions to prevent deficiency in a range of at risk groups and regions of the world.

## 1. Introduction

Vitamin C (ascorbic acid) is an essential nutrient that must be consumed on a regular basis to prevent deficiency [1]. Maintenance of the body pool of vitamin C is dependent on dietary intake, efficient absorption, recycling and renal reuptake of the vitamin [2]. Vitamin C concentrations in blood and tissues are tightly controlled via specialized sodium-dependent vitamin C transporters (SVCTs) [3]. Different tissues and organs have variable requirements for vitamin C, as reflected by their vitamin C concentrations [4]. Tissues with the highest concentrations of the vitamin include the brain, adrenals and pituitary gland. This reflects one of the major functions of vitamin C, which is to act as a cofactor for a family of biosynthetic and regulatory metalloenzymes, including those involved in the synthesis of catecholamine and peptide hormones [5,6]. Recent research has also indicated a role for vitamin C in genetic and epigenetic regulation via enzymes that regulate gene transcription and the methylation of DNA and histones [7,8]. As a result, vitamin C has the potential to regulate thousands of genes in the body and thus play pleiotropic roles in human health and disease.

Historically, recommended intakes of micronutrients have been based on daily intakes required to prevent disease secondary to deficiency. Previous reviews of micronutrients have indicated high rates of global deficiency [9]. Due to a growing body of evidence that increased vitamin C intake has a beneficial effect on long-term health outcomes [1], many regulatory authorities have increased recommended intakes of vitamin C in their respective countries [10]. Vitamin C intakes of 100–200 mg/day will maintain blood concentrations at adequate to saturating status i.e., 50–75 µmol/L [11]. When blood concentrations fall to the hypovitaminosis C range (i.e., <23 µmol/L), symptoms of vitamin C insufficiency may become apparent, such as fatigue, lethargy and mood changes, e.g., irritability and depression [11]. People with hypovitaminosis C are at high risk of developing vitamin C deficiency (defined as <11 µmol/L), putting them at risk of developing clinical scurvy, which is fatal if left untreated [12].

Global vitamin C status and prevalence of insufficiency has never been fully reported, despite increasing concern over micronutrient malnutrition in low-middle income countries (LMICs) resulting in poor health and higher rates of morbidity and mortality [13,14]. Here we provide an overview of global vitamin C status and prevalence of insufficiency and the associated potential public health impact of deficiency.

## 2. Selection and Assessment of Vitamin C Status Publications

For the illustrative purposes of this review, we describe key papers reporting the status of predominately healthy or randomly selected groups globally. A thorough literature search was conducted using the PubMed database using the keywords: vitamin C, ascorbic acid, ascorbate, blood, plasma, serum, concentration, level, status. No restrictions were placed on the publication date, study location, sex or age of participants. Further literature was found within the reference lists of published papers. Original English language publications containing plasma or serum vitamin C concentrations of healthy adults, pregnant women, children and adolescents are described. The vitamin C status of specific patient groups is not described. Countries were categorized as high, upper middle, lower middle or low income based on the World Bank income classifications [15]. Due to the scarcity of research in many LMICs smaller studies from LMICs with a minimum of 90 individuals are described. Where multiple large studies were available, e.g., USA, the most recent and largest studies are described. Where available, mean vitamin C concentrations (µmol/L) and mean dietary intakes (mg/day), rates of hypovitaminosis C and frank deficiency (%), are described. Currently, there are no internationally accepted cut off values to define hypovitaminosis C and vitamin C deficiency. Therefore, the most commonly used values have been used, e.g., ≤23–28 µmol/L for hypovitaminosis C and ≤11 µmol/L for deficiency. All plasma and serum concentrations are provided as mean and SD (or IQR) in μmol/L (converted from mg/dL or μg/mL as required) and missing data were calculated using weighted means.

## 3. Global Vitamin C Status and Prevalence of Insufficiency

### 3.1. High Income Countries

Several large epidemiological studies assessing vitamin C status and/or prevalence of deficiency in adults have been carried out in Europe and North America (Table 1). The largest study to have measured vitamin C status globally is the European EPIC-Norfolk study carried out in England (1993–1997) [16,17]. This study assessed >22,400 participants (aged 40–79 years), revealing a weighted mean of 54 µmol/L vitamin C (48 and 59 µmol/L for men and women, respectively), and a prevalence of deficiency of 1.4% (2.2% for men and 0.8% for women). Dietary intakes of vitamin C, determined using 7-day food diaries, were 85 mg/day for the cohort (83 and 87 mg/day for men and women, respectively). The smaller National Diet and Nutrition Survey carried out in 1994/1995 in the UK (England and Scotland) in >1300 elderly participants (aged ≥65 years) showed a lower vitamin C status (44 ± 25 µmol/L), and a higher prevalence of deficiency (14%) [18], more comparable to the French POLA study of the same age group (see below). The third MONICA study, carried out in Glasgow, Scotland in 1992 in >1200 adults, indicated an even higher prevalence of deficiency of 20% for the cohort (26% for men and 14% for women) [19].

The SU.VI.MAX study carried out in France (1994/1995) assessed factors influencing blood concentrations of antioxidant vitamins in >12,700 French participants aged 35–60 years [20,21]. This study showed comparable results to EPIC-Norfolk, with the vitamin C status of men and women being 50 ± 23 and 60 ± 31 µmol/L, respectively, giving a weighted mean of 56 µmol/L for the total cohort. Of these, only 1% exhibited vitamin C deficiency, higher for men (1.8%) than women (0.4%). Dietary intakes of vitamin C were assessed from six 24-h dietary records during the first 18 months of the study, giving a weighted mean of 100 mg/day for the cohort (103 and 98 mg/day for men and women, respectively). Another smaller study was carried out at a similar time (1995–1997) in Sète in the South of France in >1900 elderly participants aged >60 years (the POLA study) [22]. This study indicated a lower mean vitamin C concentration of 36 µmol/L (32 and 40 µmol/L for men and women, respectively) and a higher prevalence of deficiency (9%; 13% for men and 6% for women). Other smaller studies carried out in France (Paris and Nancy) have shown intermediate vitamin C status between these two studies, i.e., 39–41 µmol/L for men and 53 µmol/L for women [23,24]. The men in Nancy had a correspondingly lower dietary intake of 80 mg/day, determined using a dietary questionnaire that collected data on the participant’s diet history during the previous year [24].

Two studies have been carried out in Finland: one in North Karelia (1992–2002) in >1600 adults aged 25–64 years [25,26] and one in Eastern Finland (1984–1989) in >1600 men aged 42–65 years [27]. The mean vitamin C status for men ranged from 37 to 48 ± 23 µmol/L, with a prevalence of deficiency of 2.2% to 5.7% [25,26,27]. A small study of apparently healthy elderly participants in the Alicante province of Spain in 2000/2001 indicated comparable vitamin C status, with 38 ± 20 µmol/L in men and 51 ± 18 µmol/L in women, giving a weighted mean of 45 µmol/L [29]. The participants had relatively high intakes of vitamin C, assessed using a semiquantitative food frequency questionnaire; 125 ± 64 mg/day for men and 136 ± 70 mg/day for women. Another small study of five countries across Europe (including Spain, France, Netherlands, Northern Ireland and Republic of Ireland) indicated a weighted mean in healthy non-smoking adults (aged 25–45 years) of 59 µmol/L vitamin C (54 and 64 µmol/L for men and women, respectively) [30]. Similar values were recorded in Graz, Austria (1991–1994); 58 ± 21 µmol/L for a cohort of 786 adult men and women (50 ± 20 µmol/L for men and 64 ± 19 µmol/L for women) [28]. In Europe, the highest vitamin C values reported to date have come from the GISELA study in Giessen Germany (1994–2004), in which independently living senior citizens had a weighted mean of 71 µmol/L vitamin C (62 and 76 µmol/L for men and women, respectively) [31]. Dietary intakes, assessed using a 3-day estimated dietary record, revealed a mean intake of 90 mg/day for the cohort (84 and 93 mg/day for men and women, respectively). The researchers attributed the lower vitamin C plasma concentrations in men compared with women partly to a volumetric dilution effect due to differences in fat-free mass.

In the United States, the National Health and Nutrition Examination Surveys (NHANES) have been reporting nationally representative vitamin C status data over nearly four decades [32,40,41]. The most recent survey (2003/2004) included >4400 adults (aged ≥20 years) and indicated a mean status of 49 µmol/L vitamin C for the cohort (45 and 53-µmol/L for men and women, respectively) [32]. The prevalence of deficiency was 8.4% (10% for men and 6.9% for women), significantly higher than that observed in the larger European SU.VI.MAX and EPIC-Norfolk studies. Dietary intakes were not reported in this study, however, the earlier NHANES III (1988–1994) report indicated a mean dietary intake of ~106 mg/day (determined using 24-h dietary recalls) for a similarly aged cohort (≥18 years) [41]. The recent Canadian Health Measures Survey (2012/2013) assessed the vitamin C status of >1600 adults (aged 20–79 years) and reported a mean vitamin C status of 53 µmol/L (47 and 59 µmol/L for men and women, respectively). The prevalence of deficiency was <3%, in line with the large European studies (SU.VI.MAX and EPIC-Norfolk). Another smaller study was carried out in >900 young non-smoking Canadians (aged 20–29 years), however, the vitamin C concentrations were unusually low (31 µmol/L, with 14% deficiency), despite relatively high dietary intakes of 242 mg/day for the total cohort (140 mg/day for non-supplement users), assessed using food frequency questionnaires for dietary intake over the previous month [34]. The low vitamin C status in this study has been attributed to the blood samples not being processed or stored appropriately for accurate vitamin C analysis [42,43].

Several studies have been carried out in high-income countries in Asia and the Pacific (Table 1). In Japan, the largest study was undertaken in 1977 (in Shibata, Niigata Prefecture) [35]. This study comprised >2100 adults aged 40–89 years and indicated a mean vitamin C status of 51 µmol/L (43 ± 19 µmol/L for men and 57 ± 17 µmol/L for women). A more recent study carried out in Tokyo (2006) in >600 elderly women (aged 70–84 years) indicated a comparable plasma vitamin C status of 51 ± 9 µmol/L. Middle-aged participants in the South Island of New Zealand had a mean vitamin C status of 44 µmol/L (41 and 47 µmol/L for men and women, respectively) and a relatively low prevalence of deficiency of 2.4% (4.0% for men and 1.0% for women). The average dietary intake of these participants was 110 mg/day (113 and 107 mg/day for men and women, respectively), determined using four day estimated food diaries [39]. In contrast, the vitamin C status of adults (aged 30–69) in Singapore was lower at a mean of 37 µmol/L (32 and 41 µmol/L for men and women, respectively), with 12% deficiency (17% for men and 6% for women) [37,38]. Lower vitamin C concentrations were reported in Indians and Malays than Chinese in this study. The authors suggested this may have been due to low intakes of fresh fruit and different traditional cooking practices and cuisines.

### 3.2. Low and Middle Income Countries

There have been few vitamin C status studies carried out in Central and South America (Table 2). Two studies in Mexican women have indicated low vitamin C status (19 and 30 ± 13 µmol/L) and high rates of hypovitaminosis C and deficiency (up to 32% and 39%) [44,45], despite reported mean dietary intakes of 72 mg/day, based on three 24-h recalls [44]. Hypovitaminosis C was associated with obesity in one of the studies [44]. Regional variations were seen with lower deficiency rates in Mexico City than North and South Mexico [45]. A study of 369 elderly people of low socioeconomic status in Quinto, Ecuador indicated a very low mean vitamin C status of 15 µmol/L (11 ± 9 µmol/L for men and 17 ± 10 µmol/L for women) and a high prevalence of deficiency (43%), with men exhibiting more deficiency than women (60% vs. 33%, respectively) [46]. Another small study of 117 pregnant females admitted to hospital in Sao Paulo, Brazil, showed a mean vitamin C status of 33 µmol/L and 31% to have hypovitaminosis C [47].

A limited number of studies have been performed assessing vitamin C status across Africa (Table 2), none of which are large scale epidemiological studies of the general population, having primarily investigated specific groups. In South Africa a small study assessed vitamin C concentrations in 285 elderly participants [48]. A low mean vitamin C status of 25 µmol/L was observed in these participants (23 and 25 µmol/L for men and women, respectively), and was reflected by low vitamin C intakes (mean 39 mg/day; 27 and 42 mg/day for men and women, respectively). A high prevalence of hypovitaminosis C (defined as <34 µmol/L in this study) was observed (mean 66%; 84% for men and 62% for women). A number of small studies have been carried out in Nigerian women. One study of 400 antenatal clinic patients showed low vitamin C status (20 ± 29 µmol/L) with 80% of the women exhibiting hypovitaminosis C [49]. The authors attributed this to high parity, inadequate nutrition and nutritional taboos among Northern Nigerian females. Of note, a smaller study of female hospital and university staff in South Nigeria during the rainy season reported very high mean vitamin C intakes (>680 mg/day, determined by 24-h diet history), saturating vitamin C status (74 µmol/L), and no cases of hypovitaminosis and deficiency were seen [50]. In Uganda, research into pre-eclampsia in Kampala (Malago Hospital), 400 well pregnant women were studied and showed very low vitamin C status (only 11 ± 4 µmol/L) and high rates of vitamin C deficiency (70%) [51]. Additionally, a control group of 200 well women from clinics also showed low vitamin C status (15 ± 6 µmol/L) and 28% hypovitaminosis C. The differences between these studies may be a reflection of seasonal variation and disparities in intake across socioeconomic and regions of the continent, or limitations of the assay methodologies used. Of note, clinical outbreaks of scurvy still occur in Africa such as a recent outbreak in tribesmen in Kenya [52].

Vitamin C deficiency in India has been relatively well characterized with a large population-based study (Table 2). In this study, of >5600 adults aged over 60, frank deficiency was observed in 74% of adults in North India and 46% of adults in South India, with a higher prevalence of deficiency observed in men than women [57]. This was reflected by low dietary intakes of vitamin C (determined by 24-h dietary recall) of 23 and 34 mg/day for North and South India, respectively. Very low mean vitamin C concentrations of 15 µmol/L (13 and 17 µmol/L for men and women, respectively) in villages in North India have been reported [58]. A small study of healthy adults in Western India showed comparably low vitamin C status with a mean of 18 µmol/L (17 ± 7 µmol/L for men and 20 ± 7 µmol/L for women), along with high rates of deficiency and >70% hypovitaminosis C [59]. Dietary intakes of vitamin C were low (mean 34 mg/day; 40 mg/day for men and 29 mg/day for women), as determined using a food frequency questionnaire of usual dietary intake over the previous year.

Mean vitamin C concentrations are available from several studies in China (Table 2). One in Linxian indicated relatively low mean concentrations of 33 µmol/L for people aged ≥50 years (27 and 37 µmol/L for men and women, respectively) [54]. A higher mean status of 45 µmol/L was observed in >2000 women aged 30–64 years in Shanghai [53]. In contrast, women in the third trimester of pregnancy had a low mean vitamin C status of 19 µmol/L and a high prevalence of hypovitaminosis C (66%) [55]. Research in Russia has shown extremely low mean vitamin C status (9 µmol/L), particularly in men, with high rates of deficiency (79%) and hypovitaminosis C (90%) in adult males [25,26]. A small study in Bangkok, Thailand, indicated a low mean vitamin C status (36 µmol/L) and high prevalence of hypovitaminosis C (31%) [56]. Similar values were reported in Java, Indonesia, with a mean status of 29 ± 19 µmol/L and 45% hypovitaminosis C [60]. Elsewhere in Asia seasonal severe outbreaks of clinical scurvy have been noted in the winter months in Afghanistan occurring at a prevalence rate of 6.3% towards the end of the winter months [61].

### 3.3. Children and Adolescents

Although vitamin C pharmacokinetics are relatively well understood in adult men and women [11,62], relatively little is known about its pharmacokinetics in children and adolescents. However, based on its newly discovered epigenetic roles, vitamin C may be particularly important for the growth and development of infants and children [63]. Global recommended intakes for children and adolescents are less than adults and are generally based on their lower body weight [10]. In well-nourished populations, such as the USA, the vitamin C status of children is higher (mean 71 µmol/L) and the prevalence of deficiency lower (1.6%) than adults [32]. However, this is not necessarily the case in LMIC, where low vitamin C status has been observed in children and adolescents (Table 3). Studies in Mexico have shown high rates of deficiency and hypovitaminosis C in children [45,64]. Mean vitamin C concentrations in school aged children were low at 28 and 24 ± 9 µmol/L, with up to 38% hypovitaminosis C and 23% deficiency. A food frequency questionnaire indicated a mean intake of 44 mg/day vitamin C [64]. Of note, rates of overweight and obese children in the more recent Mexico study were high at 44%, and vitamin C concentrations were inversely associated with body fat and abdominal fat [64].

Several studies have been carried out in children of different ages in India. Malnourished preschool children in Jaipur city were shown to have a higher prevalence of deficiency than well-nourished children [65]. The low prevalence of deficiency of this cohort (1.1%) is possibly due to the young age and consequently lower body weight of these children. In contrast, a high prevalence of 60% hypovitaminosis C (defined as <30 µmol/L) has been observed in older children in Hyderabad [66]. Poor vitamin status (43 ± 26 µmol/L) and a high prevalence of hypovitaminosis C (34%) has been observed among adolescent girls in the slums of Delhi [67]. They reported a mean dietary intake of 48 ± 26 mg/day, as determined by 24-h dietary recall. A study from Bangladesh demonstrated a comparable mean vitamin C status of 46 ± 20 µmol/L in rural adolescent females (with mild to moderate anemia), however, only 11% had hypovitaminosis C [68]. This is likely due to the study being carried out during the rainy season during which time green leafy vegetables were likely to have been widely available. In a small study in Nigeria, male and female adolescents were found to have very low dietary intakes, determined by a 3-day weighed food intake in the boarding students, and correspondingly low vitamin C status [69]. Nearly 50% of the adolescents had vitamin C levels <40 µmol/L (60% in boys and 40% in girls).

**Figure 1:**
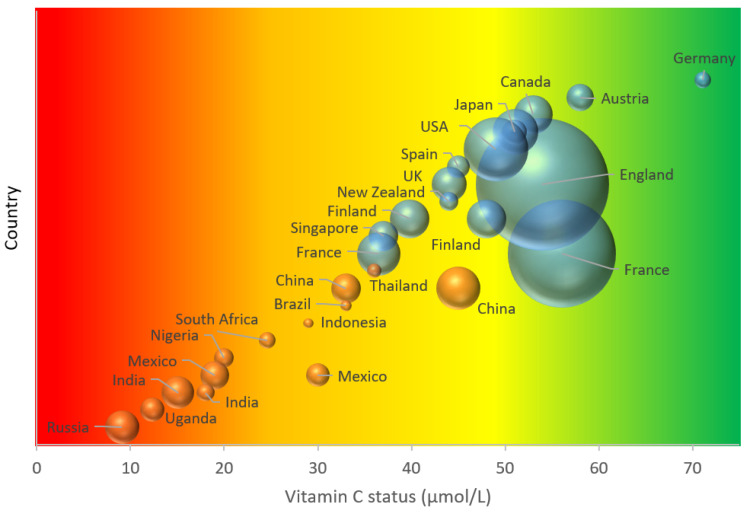
Global adult vitamin C status. The area of the bubble represents the size of the study. Blue bubbles represent high-income countries; orange bubbles represent low- and middle-income countries. Vitamin C status cutoffs: red—deficient (<11 µmol/L); orange—hypovitaminosis C (<23 µmol/L); yellow-inadequate (<50 µmol/L); green—adequate (>50 µmol/L).

**Figure 2:**
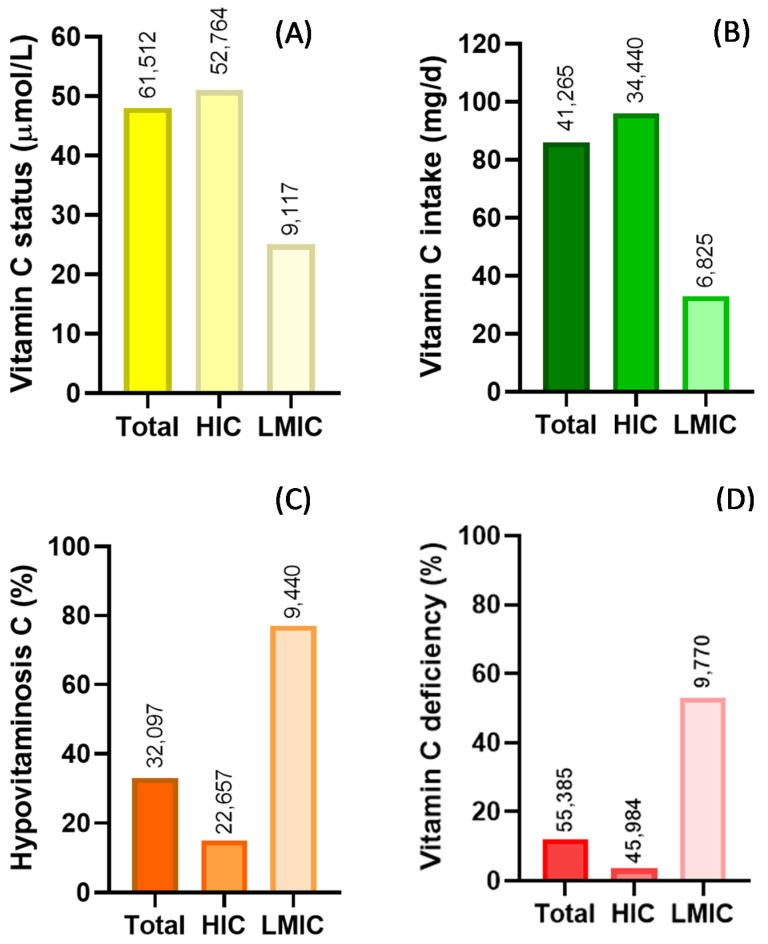
Summary of global vitamin C status (**A**) and intake (**B**) and prevalence of hypovitaminosis C (**C**) and vitamin C deficiency (**D**). Key: HIC—high-income countries; LMIC—low- and middle-income countries. Hypovitaminosis C, <23 µmol/L; vitamin C deficiency, <11 µmol/L. Numbers above bars indicate total number of individuals assessed.

Overall, there are clear disparities in vitamin C status and prevalence of deficiency between high-income countries and LMICs. This is illustrated in Figure 1. There are numerous factors that likely contribute to the observed disparities between the various populations, with differences in dietary intakes between high-income countries and LMIC likely playing a major role (Figure 2). Dietary intakes can be influenced by geographic, economic, social and cultural factors [70]. Many staple foods, particularly those that are grain-based, contain negligible vitamin C. Furthermore, various processing during food preparation and traditional cooking practices employing longer cooking times, can deplete the vitamin C content of food [71], and these are not typically accounted for in assessments of vitamin C intake.

## 4. Limitations of Vitamin C Status Studies

The assessment of plasma vitamin C status comes with a number of limitations. Due to the sensitivity of vitamin C to oxidation, appropriate handling, processing and storage of biological samples prior to analysis is very important for accurate determination of the vitamin [43]. Vitamin C status can be measured using a range of methods, many of which have limitations [72]. The current gold standard is HPLC analysis, however, a number of other cheaper spectrophotometric methods are available and used globally; these can be prone to interference by other compounds in the samples [72]. All of these analytical methods require meticulous attention to technique to prevent oxidation and loss of vitamin C. These methodological issues may be problematic in LMIC due to lack of adequate infrastructure and resources, particularly in rural settings, which could result in falsely low levels and as such should be interpreted with some caution. Nevertheless, assessing circulating vitamin C concentrations may be a more accurate indicator of status than dietary intake estimates alone as these have numerous methodological issues, e.g., recorder bias, which can result in an overestimate of vitamin intakes [73]. Intakes are also frequently calculated by nutritional tables that rarely factor in the effects of storage, processing and cooking on vitamins, which contribute to overestimation of intakes. Cassava, a major component of the staple diet in many LMICs, is an example of this where almost 100% of the vitamin C is lost prior to consumption [71]. The studies presented in Table 1, Table 2 and Table 3 have used a variety of dietary assessment tools, from 24-h dietary recalls to food frequency questionnaires of various durations. It is also sometimes unclear whether supplemental intakes of the vitamin have been included or excluded in the reported intakes.

Mean vitamin C status is commonly reported, which is not necessarily a good indicator as population distributions can mask significant rates of deficiency. A further issue is consensus on the cutoffs for inadequacy; vitamin C deficiency is usually defined as plasma concentrations <11 µmol/L, however, although hypovitaminosis C is often defined as <23 µmol/L, some studies report ‘inadequacy’ as <28 µmol/L (or even <30 or 34 µmol/L), therefore making it difficult to directly compare prevalence between studies. Vitamin C ‘adequacy’ has been more recently defined as >50 µmol/L [74], however, many of the older studies report concentrations >28 µmol/L as being ‘adequate’. Because plasma vitamin C status is affected by recent dietary intake, particularly in replete people [75], assessment of fasting samples is preferred. However, this is not always possible to do, and some studies do not state whether fasting samples were analyzed. Nevertheless, in populations that are predominantly hypovitaminosis C, non-fasting is less likely to be an issue due to depleted body stores moderating fluctuations in plasma levels following consumption of the vitamin. Supplement intake also affects status [33], however, whether supplement use has been included or excluded in the study participants is not always reported.

A significant number of the large epidemiological studies were carried out in the 1990s and early 2000s, thus, much of the vitamin C status data are now ≥20 years out of date. Therefore, in light of increases in obesity and related chronic cardiometabolic health conditions such as diabetes and cardiovascular disease worldwide the results must be extrapolated with caution. Furthermore, many of the studies have been carried out in limited or defined regions with very few being nationwide and randomly selected. Therefore, most are not representative of the countries as a whole, particularly as status has been demonstrated to vary significantly based on geographic region. Finally, only a small percentage of the world’s countries have been represented, most of which were high- and upper middle-income countries. This is of concern since there are clearly significant issues with vitamin C status and prevalence of deficiency in LMIC.

## 5. Associations between Vitamin C Status and Health

There are numerous factors that affect vitamin C status and requirements, including health aspects such as obesity; these have been covered in detail in other recent reviews [10,76]. Low vitamin C status is thought to be both a cause and a consequence of various communicable and non-communicable diseases. Lower vitamin C status has been observed in pre-diabetes and metabolic syndrome, indicating that depletion of the vitamin begins prior to development of the cardiometabolic disorders diabetes and cardiovascular disease [77,78]. Numerous epidemiological studies have indicated an association between vitamin C intake or status and overall mortality, including mortality due to cardiovascular disease and malignancies [79,80,81]. Vitamin C could simply be a marker for healthy fruit and vegetable consumption, however, it appears to have a stronger association with cancer and cardiovascular disease mortality than other micronutrients [80]. It is important to note that many prospective vitamin C intervention studies of non-communicable diseases have been carried out in predominantly replete populations, thus precluding an effect of the intervention and resulting in equivocal findings [82]. The interventional studies of vitamin C supplements or dietary interventions with positive outcomes have predominantly been in groups at high risk of insufficiency, such as institutionalized individuals in Europe [83].

The vitamin has a number of mechanistic rationales for its pleiotropic roles in human health and disease, including both antioxidant and cofactor functions [1,84]. For example, the particularly high concentrations of vitamin C in the adrenal and pituitary glands indicate important roles in hormonal regulation and the stress response [6]. Its high concentrations in the brain and associations with cognitive function also indicate important roles in the functioning of the central nervous system [39,85]. Vitamin C is widely accepted to play important roles in optimal immunological function [86]. Vitamin C deficiency has been associated with respiratory infections such as pneumonia, which is also one of the major complications and causes of death in cases of scurvy [87]. Leukocytes of both the innate and adaptive immune systems contain high concentrations of the vitamin and it is believed to play important roles in their development and immune functions [86]. While the optimum intake remains unclear, a healthy intake of at least 200 mg/day may be protective against a wide range of globally important infectious diseases [87,88]. Enhanced intakes could also potentially prevent less severe infections from developing into more severe conditions, such as sepsis, which is a major cause of communicable disease morbidity and mortality worldwide [88]. This is of particular relevance with regard to the current coronavirus (SARS-CoV-2) pandemic [89].

## 6. Conclusions and Future Directions

With the above limitations in mind, this review indicates that vitamin C deficiency is likely to be common globally—and particularly so in low-income groups and low-middle income countries. Despite this, the vitamin C status of many countries and populations has not yet been assessed. Therefore, further research is required to explore this in many regions of the world; to do this accurately will require improved methods for sampling, processing, storing and analyzing samples. Currently, point-of-care vitamin C monitors are in development; depending on their accuracy, these could be used directly in the field to aid in further assessment of vitamin C status globally.

Given the growing burden of non-communicable disease in low- and middle-income settings and potential protective effects of healthy vitamin C intakes against cardiovascular diseases, a range of malignancies, cataracts and multiple infectious diseases [1,84,87], improving vitamin C intake in these areas may prove to be a cheap and effective public health intervention. Despite this, recommended dietary intakes of vitamin C vary significantly between countries (Table 4), with the recommendations of some health authorities being primarily for prevention of deficiency (i.e., 40–45 mg/day), rather than optimization of health (i.e., 200 mg/day) [10]. Reassessment of vitamin C requirements has not been high on the agenda of various international health authorities, but the data indicate that it is clearly a nutrient of concern in many countries. The high vitamin C status reported in older adults in Germany, which has one of the highest dietary recommendations in the world [31,90], indicates that it is possible to achieve saturating vitamin C concentrations through the diet and/or supplementation. This calls to the need for harmonization of vitamin C recommendations globally.

Interventions to help optimize vitamin C nutriture worldwide could include: education (clinical and public, e.g., Global Nutrition and Empowerment), increased dietary recommendations (e.g., by the Food and Agriculture Organization/WHO), reduced tax on fresh fruit and vegetables, encouraging local growing of fruit and vegetables in LMICs, novel vitamin C tax reductions for products such as soft drinks that are high in vitamin C and government subsidies for supplementation (e.g., Vitamin Angels), and including regular provision to institutionalized individuals (e.g., elderly and school children). Research has indicated that the bioavailability of vitamin C from supplements is comparable to that from fruit and vegetables [91], although the latter is encouraged due to the presence of other essential nutrients and health-promoting phytochemicals. However, it is not always possible for people to obtain sufficient vitamin C through diet alone, particularly in light of the low vitamin C content of many staple foods [76]. Therefore, additional supplementation is encouraged in these situations, particularly if there are also underlying morbidities or other risk factors that increase the requirement for the vitamin [76]. Furthermore, of importance to note is that micronutrient deficiencies often do not occur in isolation, particularly in LMICs [46].

Overall, vitamin C deficiency appears common in low- and middle-income settings and not uncommon in many other settings, particularly in at risk groups. Further studies are required to confirm these findings, including in the countries not yet represented, and to fully understand the associations of vitamin C with a range of disease processes. Globally, clinicians should remain vigilant to vitamin C deficiency as a cause or contributing factor to many common presentations. Our findings also suggest a need for interventions to prevent deficiency in a range of at risk groups and regions of the world.

## Figures and Tables

**Table 1:** Vitamin C status and prevalence of deficiency and hypovitaminosis C in adults from high-income countries.

Country (Region) Sampling Dates	Population (Age Range)	Vitamin C Status (µmol/L) *^a^*	Deficiency (% <11 µmol/L)	Hypovitaminosis C (% <23 or 28 µmol/L)	Dietary Intake (mg/day) *^a^*	References
**Europe**						
England (Norfolk) 1993–1997	22,474 total (40–79 years)	54 *^b^*	1.4	12	85 *^b^*	[16,17]
	10,267 males	48 *^b^*	2.2	17	83 *^b^*	
	12,207 females	59 *^b^*	0.8	8	87 *^b^*	
UK (England and Scotland) 1994–1995	1310 elderly (≥65 years)	44 (25)	14	–	–	[18]
Scotland (Glasgow) 1992	1267 total (25–74 years)	–	20	44	–	[19]
	632 males	–	26	52	–	
	635 females	–	14	36	–	
France (all regions) 1994–1995	12,741 total (35–60 years)	56 *^b^*	1.0	–	100 *^b^*	[20,21]
	5028 males	50 (23)	1.8	–	103 (48)	
	7713 females	60 (31)	0.4	–	98 (44)	
France (South; Sète) 1995–1997	1987 total (>60 years)	36 *^b^*	9 *^b^*	–	–	[22]
	874 elderly males	32	13 *^b^*	–	–	
	1113 elderly females	40	6 *^b^*	–	–	
France (Paris) <1991	837 total (≥18 years)	48 *^b^*	–	–	–	[23]
	361 males	41	–	–	–	
	476 females	53	–	–	–	
France (Nancy)	459 males (20–60 years)	39 *^b^*	–	–	80 *^b^*	[24]
Finland (North Karelia) 1992–2002	1616 total (25–64 years)	40 *^b^*	–	–	–	[25,26]
	974 males	37	2.2	4.4	–	
	642 females	44	–	–	–	
Finland (Eastern) 1984–1989	1605 males (42–60 years)	48 (23)	5.7	–	–	[27]
Austria (Graz) 1991–1994	786 total (45–86 years)	58 (21)	–	–	–	[28]
	330 males	50 (20)	–	–	–	
	456 females	64 (19)	–	–	–	
Spain (Alicante province) 2000–2001	545 total (>65 years)	45 *^b^*	–	–	131 *^b^*	[29]
	252 elderly males	38 (20)	–	–	125 (64)	
	293 elderly females	51 (18)	–	–	136 (70)	
Europe (France, Ireland, Spain Netherlands)	349 total (25–45 years)	59 *^b^*	–	–	–	[30]
	175 males	54 (13–103) *^d^*	–	–	–	
	174 females	64 (6–117) *^d^*	–	–	–	
Germany (Giessen) 1994–2004	279 total (62–92 years)	71 *^b^*	–	–	90 *^b^*	[31]
	98 elderly males	62 (55–74) *^c^*	–	–	84 (61–116) *^c^*	
	181 elderly females	76 (62–89) *^c^*	–	–	93 (70–132) *^c^*	
**North America**						
United States 2003–2004	4438 total (≥20 years)	49 (32–71) *^c^*	8.4	–	–	[32]
	2153 males	45 (27–66) *^c^*	10.0	–	–	
	2285 females	53 (38–76) *^c^*	6.9	–	–	
Canada (16 sites countrywide) 2012–2013	1615 total (20–79 years)	53	<3	–	–	[33]
	804 males	47	–	–	–	
	811 females	59	–	–	–	
Canada (Toronto) 2004–2008	979 total (20–29 years)	31	14	47	140/242 *^b,e^*	[34]
	287 males	29	16	37	228	
	692 females	33	13	45	248	
**Asia-Pacific**						
Japan (Shibata, Niigata Prefecture) 1977	2185 total (40–89 years)	51 *^b^*	–	–	–	[35]
	919 males	43 (19)	–	–	–	
	1266 females	57 (17)	–	–	–	
Japan (Itabashi, Tokyo) 2006	655 elderly females	51 (9)	–	–	–	[36]
	(70–84 years)					
Singapore 1993–1995	941 total (30–69 years)	37 *^b^*	12 *^b^*	–	–	[37,38]
	468 males	32 *^b^*	17 *^b^*	–	–	
	473 females	41 *^b^*	6.0 *^b^*	–	–	
New Zealand (Canterbury region) 2010–2013	369 total (50 years)	44	2.4	13	110	[39]
	174 males	41	4.0	15	113	
	195 females	47	1.0	11	107	

*^a^* Data represent mean (SD), if provided; *^b^* For missing data, weighted means were calculated; *^c^* Mean or median and interquartile range; *^d^* Mean and range; *^e^* Intake without/with supplement use.

**Table 2:** Vitamin C status and prevalence of deficiency and hypovitaminosis C of adults in low- and middle-income countries.

Country (Region) Sampling Dates	Population (Age Range)	Vitamin C Status (µmol/L) *^a^*	Deficiency (% <11 µmol/L)	Hypovitaminosis C (% <23 or 28 µmol/L)	Dietary Intake (mg/day) *^a^*	References
**Upper-middle**						
Russia (Pitkäranta District, Republic of Karelia) 1992–2002	1191 total (25–64 years)	9.0 *^b^*	–	–	–	[25,26]
	579 males	5.0	79	90	–	
	612 females	13	–	–	–	
China (Shanghai) 1995–2001	2031 females (30–64 years)	45 (48)	–	–	–	[53]
China (Linxian) 1999–2000	948 total (~50–79 years)	33 (14–55) *^c^*	–	–	–	[54]
	473 males	27 (11–50) *^c^*	–	–	–	
	475 females	37 (16–57) *^c^*	–	–	–	
China (Gansu, Guangxi, Shandong, Fujian) 1999–2001	734 pregnant females (20–35 years)	19 *^b^*	–	66	–	[55]
Thailand (Bangkok) 2003	209 total (23–68 years)	36 (0–102) *^d^*	–	31 *^b^*	–	[56]
	90 males	37 (0–77) *^d^*	–	33	–	
	119 females	–		30	–	
Mexico (multiple states)	855 non-pregnant females (12–49 years)	19	39	–	–	[45]
Mexico (Central-Queretaro state) 2012	580 females (37 years) *^e^*	30 (13)	5	32	72 *^b^*	[44]
Ecuador (Quito) 2003–2004	369 total (>65 years)	15 *^b^*	43 *^b^*	–	–	[46]
	125 elderly males	11 (9)	60	–	–	
	224 elderly females	17 (10)	33	–	–	
Brazil (Sao Paulo) 2008	117 pregnant females (≥15 years)	33 (2)	6	31	–	[47]
South Africa (Cape Town) 2015	285 total (≥60 years)	25 *^b^*	–	66 *^b,f^*	39 *^b^*	[48]
	53 elderly males	23	–	84 *^f^*	27	
	232 elderly females	25	–	62 *^f^*	42	
**Lower-middle**						
India (North-Haryana state; South-Tamil Nadu) 2004–2006	5638 total (≥60 years)	–	59 *^b^*	81 *^b^*	29 *^b^*	[57]
	2668 North total	–	74	89	23 *^b^*	
	1283 elderly males	–	78	–	–	
	1385 elderly females	–	71	–	–	
	2970 South total	–	46	74	34 *^b^*	
	1407 elderly males	–	51	–	–	
	1563 elderly females	–	40	–	–	
India (North-Balba-garh, Faridabad district) 2002–2003	1112 total (≥50 years)	~15 *^b^*	–	–	–	[58]
	~48% males	13	–	–	–	
	~52% females	17	–	–	–	
India (West-Maharashtra state) 1998–2000	322 total (20–45 years)	18 *^b^*	18 *^b^*	71 *^b^*	34 *^b^*	[59]
	214 males	17 (7)	20	75	40 *^b^*	
	108 females	20 (7)	13	63	29 *^b^*	
Indonesia (West Java) 2011	98 total (39–50 years)	29 (19)	11	45	–	[60]
	45 males; 53 females					
Nigeria (Northwest-Kano state) 2009–2011	400 pregnant females (<20–39 years)	20 (29)	–	80	–	[49]
Nigeria (South-east-Enugu) 2009	200 non-pregnant females (29 years) *^b,e^*	74 *^b^*	0	0	683 *^b,g^*	[50]
**Low income**						
Uganda (Kampala) 2008–2009	600 females (15–49 years)	12 *^b^*	56 *^b^*	–	–	[51]
	400 pregnant	11 (4)	70	–	–	
	200 non-pregnant	15 (6)	28	–	–	

*^a^* Data represent mean (SD), if provided, *^b^* For missing data, weighted means were calculated, *^c^* Mean or median and interquartile range, *^d^* Median and range; *^e^* Mean age; *^f^* Cutoff of 34 µmol/L; *^g^* Converted from weighted mean of 3882 µmol using Mr of 176.

**Table 3:** Vitamin C status and prevalence of deficiency and hypovitaminosis C of children and adolescents globally.

Country (Region) Sampling Dates	Population (Age Range)	Vitamin C Status (µmol/L) *^a^*	Deficiency (% <11 µmol/L)	Hypovitaminosis C (% <23 or 28 µmol/L)	Dietary Intake (mg/day) *^a^*	References
**High income**						
United States 2003–2004	823 children (6–11 years)	71 *^b^*	1.6 *^b^*	–	–	[32]
	400 boys	74 (60–88) *^c^*	1.3	–	–	
	423 girls	69 (56–87) *^c^*	1.8	–	–	
	2016 adolescents (12–19 years)	53 *^b^*	3.3 *^b^*	–	–	
	1037 boys	51 (37–68) *^c^*	2.7	–	–	
	979 girls	55 (38–76) *^c^*	3.9	–	–	
**Upper-middle**						
Mexico (multiple states) 1999	1815 children (0–11 years)	28	23	–	–	[45]
Mexico (Queretaro state) 2012	197 children (6–11 years)	24 (9)	8	38	44	[64]
**Lower-middle**						
India (Jaipur city)	5000 well-nourished preschool children	–	0	–	–	[65]
	1000 malnourished preschool children	–	1.1	–	–	
India (Hyderabad)	869 children (6–16 years)	–	–	60 *^d^*	–	[66]
India (Delhi slum) 2012–2013	775 adolescent females (11–18 years)	43 (26)	6.3	34	48 (26)	[67]
Bangladesh (Dhaka district) 2003	307 adolescent females (14–18 years)	46 (20)	2.0	11	–	[68]
Nigeria (Enugu state)	90 adolescents (13–20 years)	–	–	47 *^e^*	–	[69]
	Males	35–43	–	60 *^e^*	24–27	
	Females	47–51	–	40 *^e^*	15–20	

*^a^* Data represent mean (SD), if provided; *^b^* For missing data, weighted means were calculated; *^c^* Mean or median and interquartile range; *^d^* Cutoff <30 µmol/L; *^e^* Cutoff <40 µmol/L.

**Table 4:** Global recommended dietary intakes for vitamin C.

Country or Authority	Males (mg/day)	Females (mg/day)
**High Income**		
France	110	110
DACH, European Union	110	95
Japan	100	100
Italy, Singapore	105	85
USA, Canada	90	75
Nordic, Netherlands	75	75
Spain	60	60
Australia, New Zealand, FAO/WHO	45	45
United Kingdom	40	40
**Low-Middle Income**		
China	100	100
South Africa	90	90
Thailand	90	75
Malaysia	70	70
Vietnam	70	65
Philippines	70	60
Indonesia	60	60
India	40	40

DACH—Germany, Austria, Switzerland; Nordic—Denmark, Finland, Iceland, Norway, Sweden; FAO/WHO—Food and Agriculture Organization/World Health Organization. Data from [10].
